# The Impact of Empathy on Prosocial Behavior Among College Students: The Mediating Role of Moral Identity and the Moderating Role of Sense of Security

**DOI:** 10.3390/bs14111024

**Published:** 2024-11-01

**Authors:** Li Peng, Yu Jiang, Jing Ye, Zhiheng Xiong

**Affiliations:** 1 School of Humanities, Southeast University, Nanjing 211189, China; pengli@seu.edu.cn (L.P.); 101011720@seu.edu.cn (Y.J.); 2 School of Electronic Science and Engineering, Southeast University, Nanjing 211189, China; yejing@seu.edu.cn

**Keywords:** college students, empathy, prosocial behavior, moral identity, sense of security

## Abstract

To investigate the impact of empathy on prosocial behavior and the underlying psychological mechanisms among college students, this study surveyed 840 participants using the Empathy Scale, the Prosocial behavior Scale, the Moral Identity Scale, and the Sense of Security Scale. The results revealed that (1) empathy significantly and positively predicted prosocial behavior among college students; (2) moral identity partially mediated the relationship between empathy and prosocial behavior; (3) a sense of security moderated the relationship between moral identity and prosocial behavior. These findings offer important theoretical and practical implications, enriching the theoretical framework and providing educators and students with valuable guidance.

## 1. Introduction

Prosocial behavior encompasses the positive and altruistic actions that individuals undertake during social interactions, such as cooperation, helping, and sharing. These behaviors are associated with several positive outcomes, including increased self-esteem, academic achievement, and enriched interpersonal relationships [1]. Previous research underscores the importance of prosocial behavior at both individual and societal levels, highlighting that it not only benefits others but also enhances the well-being of the individuals who engage in it. The role of socialization in moderating anxiety among graduate students has been demonstrated [2]. The prosocial behavior attitude of students often reflects their socialization; those who are more inclined to engage in prosocial actions are better equipped to adapt to society and enjoy greater overall well-being. Therefore, it is essential to investigate the prosocial behavior of students and the underlying influencing factors to provide a basis for implementing effective interventions that promote positive social adaptations among students.

Previous studies have shown that empathy is closely associated with prosocial behavior [3]. Empathy, defined as the ability to perceive other’s feelings and understand their intentions, plays a crucial role in fostering prosocial behavior [4]. It requires not only recognizing the emotions and needs of others but also experiencing emotional resonance and motivational transformation, which encourage proactive efforts to assist others. Although a connection between empathy and prosocial behavior has been established in previous studies, evidence regarding how the former affects the latter remains poorly explored.

Recent studies have increasing focused on the close relationship between empathy and moral identity [5,6]. When individuals develop empathy during social interactions, they may become more attentive to the needs and feelings of others, which can enhance their moral identity and further influence their behavior [7]. As the self-model theory indicates that, when individuals integrate certain moral values into their self-model, these values become part of their moral identity, affecting their decision-making and behavior [8,9]. Additionally, studies have shown a positive correlation between moral identity and mental health, indicating that these positive psychological states may be important components of a sense of security [10]. A sense of security refers to the feeling of being safe, stable, and free from fear or anxiety. According to emotional security theory (EST), children’s behavioral adjustment is linked to their emotional security. Moreover, an empirical study demonstrated that nursing students’ risk perception can impact their professional commitment [11]. Therefore, a sense of security may moderate the relationship between moral identity and prosocial behavior, enhancing an individual’s motivation to engage in actions that benefit others. However, based on a review of the current literature, it seems that no study has examined the interaction mechanisms between empathy, moral identity, a sense of security, and prosocial behavior.

Consequently, this study aims to explore the internal mechanisms through which empathy influences prosocial behavior. A moderated mediation model was employed to examine whether, and how, moral identity and a sense of security are linked to empathy and prosocial behavior. This research contributes to the existing literature in three ways. First, it expands the theoretical framework concerning the impact of empathy on prosocial behavior, explaining the link between empathy and prosocial behavior. Second, it revealed the underlying mechanisms of the impact of empathy on prosocial behavior, moral identity, and a sense of security. Third, the model proposed in the current study provides insights into intervention strategies for enhancing prosocial behavior.

## 2. Literature Review

### 2.1. The Impact of Empathy on Prosocial Behavior

Empathy can be defined as the capacity to naturally adopt others’ perspectives, understand their feelings, and respond appropriately to their own emotional states [12]. Studies have found that individuals with high levels of empathy are more likely to engage in cooperative and helpful behaviors in response to others’ needs [13]. Empathy is positively correlated with the quality of interpersonal relationships and levels of mental health, playing an important role in social adaptation [14]. In Hume’s moral philosophy, there is a long-standing tradition of linking empathy to prosocial behavior [15], and some empirical evidence has supported this viewpoint. For example, individuals with higher levels of empathy tend to exhibit more prosocial behaviors, enhancing their coordination and cooperation with others [16]. Additionally, a longitudinal study further confirmed that empathy positively predicts the level of prosocial behavior, with its influence persisting even one year later [17].

As a positive affective trait, empathy can motivate individuals to emotionally connect with others and alleviate their suffering, prompting them to respond actively and meet the needs of others [18]. Therefore, empathy is not only an individual affective trait but also a valuable social resource, playing an indispensable role in reducing conflicts, promoting interpersonal harmony, and maintaining social stability. Previous research has extensively explored empathy in children and adolescents and its impact on prosocial behavior, which revealed a close correlation between empathy and prosocial behavior. Nevertheless, further investigation is required to better understand the development of empathy and its relationship with the prosocial behavior of college students who are on the cusp of entering society. Therefore, we propose that empathy will positively influence prosocial behavior among college students.

**Hypothesis** **H1:** *Empathy positively predicts prosocial behavior in college students.*

### 2.2. The Mediating Role of Moral Identity Between Empathy and Prosocial Behavior

Moral identity may serve as an underlying mediating mechanism between empathy and prosocial behavior. It is a moral cognitive schema concerning self-concept and plays a crucial role in transforming moral cognition and judgment into moral behavior [8]. On one hand, there is a close relationship between empathy and moral identity [5,6]. Individuals have a keen perception of others’ emotional states and needs, which often reflects their intrinsic moral values [19]. The development of empathy in social interactions may result in individuals becoming more attentive to the needs and feelings of others, thereby enhancing their moral identity [7]. Specifically, empathy may contribute to the development of moral identity in college students by fostering emotional understanding and moral concern for others. On the other hand, moral identity, as a form of social identity, is part of an individual’s social self-schemata that provides moral support for prosocial behavior [8,20]. According to the self-model theory, an individual’s behavior is often driven by their self-concept [9,21]. Therefore, individuals influenced by moral identity may consciously adhere to social and moral norms. Empirical studies have also demonstrated that a strong moral identity can enhance an individual’s sense of responsibility and intrinsic motivation, leading them to take actions consistent with their moral principles, thereby promoting prosocial behavior [22].

Therefore, it is reasonable to posit that moral identity may mediate the relationship between empathy and prosocial behavior. Specifically, moral identity can be measured through two dimensions: moral identity internalization and moral identity symbolization [8]. Moral identity internalization reflects the degree to which moral traits are integral to an individual’s self-concept, while moral identity symbolization pertains to how these traits are expressed publicly through actions. Individuals with high levels of empathy are likely to exhibit strong moral identity internalization, resonating with others’ emotions. This resonance is subsequently reflected in moral identity symbolization demonstrating a heightened concern for others. Furthermore, they may adhere to elevated moral principles, thereby encouraging them to engage in prosocial behavior when presented with requests for assistance from others. Based on this rationale, we propose the following hypothesis:

**Hypothesis** **H2:** *Empathy significantly predicts prosocial behavior through the mediating role of moral identity.*

### 2.3. The Moderating Role of Sense of Security Between Moral Identity and Prosocial Behavior

A sense of security refers to the feeling of being capable of accepting and confronting potential risks, often manifesting as a sense of certainty and control [23]. When exploring the relationship between moral identity and prosocial behavior, the sense of security may play an important moderating role. Despite the lack of direct evidence on the relationship between moral identity and sense of security, some indirect evidence has supported this potential connection. For example, individuals with a strong moral identity are more likely to adhere to moral norms and demonstrate a heightened social responsibility [24], and a sense of security tends to develop within this positive self-perception. Additionally, existing research has revealed a positive correlation between moral identity and mental health, indicating that these positive psychological states could potentially be important components of a sense of security [10].

According to emotional security theory (EST), an individual’s emotional sense of security has an important impact on their behavioral patterns and psychological processes [25]. Individuals with a strong sense of security tend to adopt a positive and trusting attitude towards others and their surroundings, and believe in their ability to cope with challenges and receive necessary support. This psychological state encourages them to follow moral principles more confidently and express and adhere to their moral beliefs more actively. In social interactions, this sense of security can enhance individuals’ motivation to engage in prosocial behavior [26]. In contrast, individuals with low levels of a sense of security are more likely to worry about external evaluations, leading to the suppression of their moral identity. They may be apprehensive about being misunderstood or excluded socially in real situations, which could result in lower self-assessments and self-protection [27].

However, with the development of moral identity, even college students with a low sense of security can enhance their prosocial behavior because they believe these actions are socially recognized and align with moral norms. Therefore, we propose that sense of security could be a moderator between moral identity and prosocial behavior.

**Hypothesis** **H3:** *Moral identity significantly predicts prosocial behavior through the moderating effect of sense of security.*

### 2.4. The Present Study

**Figure 1:**
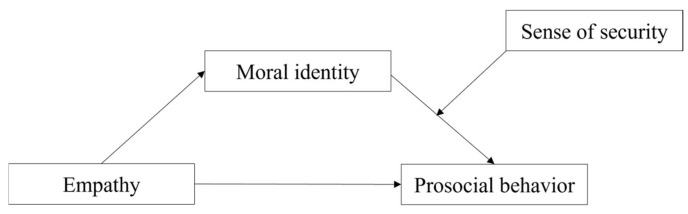
The proposed moderated mediation model.

This study aims to investigate the underlying mechanisms of empathy and prosocial behavior among college students. Specifically, we developed a moderated mediation model to test the following questions:

(1) Does moral identity mediate the relationship between empathy and prosocial behavior?
(2) Does a sense of security moderate the relationship between moral identity and prosocial behavior (see Figure 1)?

## 3. Method

### 3.1. Participants

An introductory section was included in the questionnaire to ensure that respondents understood the purpose of the study, the reason for collecting survey information, and the specific requirements for completing the questionnaire. Informed consent was obtained from all participants prior to the survey. After the survey was completed, we reviewed all data and excluded questionnaires with too short a response time (less than 180 s) and consistent responses to all questions. A total of 877 college students from two universities in Jiangsu and Heilongjiang Province, China, were recruited through convenience sampling. After deleting 37 invalid questionnaires, including those answers with omissions, errors, and regularity, 840 valid questionnaires (95.78%) were finally obtained from 144 male and 696 female students aged 17–23 years old (*M* ± *SD* = 19.41 ± 0.93). The present study was approved by the Research Ethics Committee of the School of Humanity, Southeast University.

### 3.2. Research Tools

#### 3.2.1. Basic Empathy Scale in Adults (BES-A)

The 6-item emotional contagion and 8-item cognitive empathy subscales of the Basic Empathy Scale in Adults were used to measure empathy in this study [28]. The two subscales are scored using a 5-point Likert scale ranging from 1 (strongly disagree) to 5 (strongly agree). The two dimensions are added together to calculate the total score, a higher total score indicates a higher level of empathy. In this study, the Cronbach’s α coefficient of the scale is 0.88.

#### 3.2.2. Prosocial Behavior Scale

The Prosocial Behavior Intention Scale was used for the measurement of prosocial behavior of our participants [29]. This scale consists of 4 items, scored on a 7-point Likert scale ranging from 1 (definitely would not do this) to 7 (definitely would do this). A higher total score indicates a higher level of prosociality. This scale has demonstrated a good reliability and validity among college students in China [30]. The internal consistency of this scale was high (i.e., 0.95) in the current study.

#### 3.2.3. Moral Identity Scale

The participants’ moral identities in our study were measured using the Moral Identity Scale [8]. This 10-item scale includes two dimensions: internalization and symbolization, with 5 items each. It is scored using a 5-point Likert scale ranging from 1 (strongly disagree) to 5 (strongly agree). Higher scores indicate greater levels of moral identity. This scale has previously been applied to a sample of Chinese college students and has shown good reliability and validity [31]. The Cronbach’s α coefficient of the scale in this study is 0.87.

#### 3.2.4. Sense of Security Scale

The Sense of Security Scale was used to measure our participants’ sense of security [23]. This 16-item scale consists of two subscales, including interpersonal security and certainty in control, with 8 items each. It is scored using a 5-point Likert scale ranging from 1 (extremely true for me) to 5 (not at all true for me). Higher scores indicate that a higher sense of security is experienced by the individual. This scale has demonstrated a good reliability and validity in Chinese adolescents [32]. In this study, the Cronbach’s α coefficient of this scale is 0.95.

### 3.3. Statistical Analysis

All statistical analyses were performed using SPSS 26.0 and Process 3.3. SPSS 26.0 was used for data entry, collection, descriptive statistical analysis, and correlation analysis. After calculating bivariate correlations, Hayes’ PROCESS macro Model 4 was used to test the mediating role of moral identity between empathy and prosocial behavior [33]. The moderating effect of sense of security on the second path of the mediation process was explored using Hayes’ PROCESS macro Model 14 [33]. Before applying Models 4 and 14, standardized scores for all of the variables were computed, along with interaction terms calculated from the standardized scores. Bootstrapping was used to test confidence intervals, with a 95% confidence interval (CI) calculated through 5000 repeated samples. The mediation effect refers to the relationship between variables; X → Y is an indirect effect through intermediate variable M, and this indirect causal relationship is called the mediation effect [34]. The moderation effect refers to the moderating effect of variable U when the size or positive and negative directions of the correlation between variables X and Y are influenced by variable U [34].

## 4. Results

### 4.1. Common Method Deviation

The study data collected via questionnaires may be susceptible to common method bias; therefore, Harman’s single-factor test was used to check for this bias [35]. The unrotated factor analysis results showed that there were six factors with eigenvalues greater than 1. The total variance explained by the first factor was below the critical value of 40%, indicating that there was no severe common method bias in this study.

### 4.2. Descriptive Statistics and Correlation Analysis

As shown in Table 1, empathy, prosocial behavior, moral identity, and sense of security among college students were positively correlated with each other. Additionally, gender was significantly correlated with empathy, prosocial behavior, and moral identity, while age was significantly associated with moral identity. Therefore, gender and age were included as control variables in the subsequent model analyses.

### 4.3. Mediation Effect Test

To explore the mediating role of moral identity on the relationship between empathy and prosocial behavior among college students, we used Model 4 from Hayes’ PROCESS macro for SPSS to test for mediation effects [33]. The results obtained after controlling for gender and age are presented in Table 2.

The first-step results showed that empathy significantly positively predicted moral identity (β = 0.47, *p* < 0.001). The second-step results indicated that empathy significantly positively predicted prosocial behavior (β = 0.42, *p* < 0.001). The third-step results also demonstrated that empathy significantly positively predicted prosocial behavior (β = 0.15, *p* < 0.001), and the positive predictive effect of moral identity on prosocial behavior remained significant (β = 0.57, *p* < 0.001). This indicated that moral identity serves as a mediator between empathy and prosocial behavior among college students. Further, to test the indirect effect, we used the bias-corrected percentile Bootstrap method and the mediation effect was found to be 0.27 (95% CI [0.23, 0.32]), with a standard error (SE) of 0.02, accounting for 64.51% of the total effect. This indicated that moral identity has a significant partial mediation effect between empathy and prosocial behavior.

### 4.4. Test of the Moderated Mediation Model

To examine the moderating effect of sense of security on the relationship between moral identity and prosocial behavior, we used Model 14 from Hayes’ PROCESS macro for SPSS [33]. The results obtained after controlling for gender and age are presented in Table 3. The interaction effect between moral identity and sense of security on prosocial behavior was significant (β = −0.14, *p* < 0.001). This indicated that sense of security had a significant moderating effect on the relationship between moral identity and prosocial behavior.

**Figure 2:**
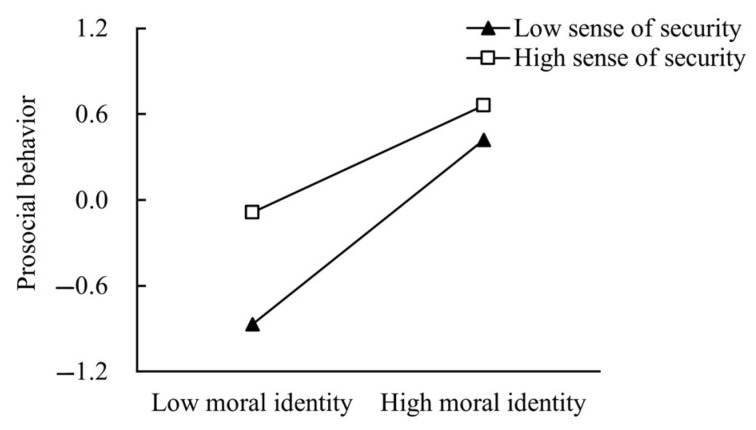
The moderating role of sense of security.

To further examine the moderating effect of sense of security on the relationship between moral identity and prosocial behavior, participants were divided into two groups according to their sense of security scores: the high sense of security group (*M* + 1*SD*) and the low sense of security group (*M*−1*SD*). The predictive effect of moral identity on prosocial behavior was then examined separately in each group. As shown in Figure 2, among college students with a low sense of security, moral identity had a significant positive predictive effect on prosocial behavior (β_simple slope = 0.64, *p* < 0.001). For college students with a high sense of security, moral identity also significantly positively predicted prosocial behavior (β_simple slope = 0.37, *p* < 0.001), but the effect was weaker. Therefore, special attention should be given to students with lower levels of sense of security, given that the positive impact of moral identity on prosocial behavior was more pronounced in this group.

## 5. Discussion

This study confirmed the positive relationship between empathy and prosocial behavior among college students, as well as the mediating role of moral identity and the moderating effect of sense of security. These findings can provide a theoretical foundation for subsequent research on prosocial behavior and play a significant role in promoting individuals’ prosocial behavior, social adaptation, and psychological health.

### 5.1. The Impact of Empathy on Prosocial Behavior

The results of this study indicated that empathy significantly positively predicted prosocial behavior among college students, which supported Hypothesis 1. Higher levels of empathy were associated with greater prosocial behavior. This finding was consistent with previous studies, highlighting the important role of empathy in promoting prosocial behavior [36,37,38]. When observing others encountering difficulties, college students with high levels of empathy are more likely to engage in prosocial behaviors, such as helping others. This finding can also be explained by the empathy–altruism hypothesis, which suggests that prosocial behaviors are motivated by the altruistic desire to alleviate others’ suffering [39]. Specifically, empathy triggers emotional fluctuations [40]. To pacify these emotions, college students may become more attentive to others’ plights and needs, thereby stimulating the motivation to help [41]. This ability enables individuals to perceive the needs and suffering of others from their own perspective, providing a strong psychological foundation for the emergence of prosocial behavior [42].

The study reveals the positive impact of empathy on prosocial behavior among college students, offering a novel perspective on the promotion of harmony and mutual assistance. To enhance prosocial behavior, college teachers should integrate empathy into mental health education, and encourage students to experience and understand the emotions and situations of others, thereby stimulating their motivation to carry out prosocial behaviors. For college students, they can enhance their empathy through interventions such as emotion regulation and active participation in community activities, which can foster their ability to think from diverse perspectives and increase sensitivity to others’ needs and difficulties, thus offering help in time.

### 5.2. The Mediating Role of Moral Identity

This study confirmed that moral identity partially mediated the relationship between empathy and prosocial behavior among college students. In particular, empathy enhanced moral identity, which in turn promoted prosocial behavior, thus supporting Hypothesis 2. This finding not only enriches the research in the field of moral psychology but also provides robust evidence of the mediating effect of moral identity between empathy and prosocial behavior. Furthermore, it offers valuable insights for educators in higher education, suggesting new approaches to promoting prosocial behavior in students. By acknowledging the role of moral identity, educators can develop strategies that nurture empathy and moral development, ultimately encouraging more prosocial actions among students.

In addition to the overall mediation mechanism, each stage of the mediation process also deserves attention. Regarding empathy and moral identity, the findings supported the notion that empathy may be a potential driving force of moral identity [43,44]. In the process of developing empathy, college students are able to understand others’ emotions, needs, and situations more deeply [45]. This emotional resonance promotes the internalization of social moral norms, transforming moral behavior into a conscious action that arises from one’s inner identity rather than external constraints [46,47]. Additionally, cognitive neuroscience research has found that empathy is closely related to brain regions that activate emotions and cognition, which are also involved in moral judgment and decision making [48,49]. These studies suggest that individuals with strong empathy are more likely to demonstrate a higher sense of moral responsibility during moral judgments, thereby enhancing their moral identity.

Regarding the relationship between moral identity and prosocial behavior, the results showed a significant positive correlation, consistent with previous research [50,51]. When individuals empathize with others’ joy or suffering, the emotional resonance can trigger moral emotions within themselves, such as compassion, pity, or a sense of justice [52,53]. These moral emotions, in turn, drive them to take prosocial actions to alleviate others’ suffering. This aligns with the social cognitive model, suggesting that college students with high levels of moral identity may have a stronger internal sense of moral responsibility [8]. In social situations, they are more likely to consider actions from a moral perspective and engage in prosocial behavior consistent with moral principles [54].

Therefore, college educators should give due consideration to the cultivation of moral identity in the context of mental health education for their students. Through comprehensive moral education and practical social engagement, educators can assist students in establishing correct moral ideas, thereby inspiring more prosocial behavior. Additionally, college students can participate more actively in community service or volunteer activities, applying and testing their moral identity in real-life situations.

### 5.3. The Moderating Role of Sense of Security

As postulated in our theoretical assumption, sense of security moderated the relationship between moral identity and prosocial behavior, thereby supporting Hypothesis 3. Sense of security not only positively predicted prosocial behavior but also moderated the effect of moral identity on prosocial behavior. Specifically, for college students with a lower sense of security, the connection between moral identity and prosocial behavior was stronger. This result aligns with EST, which posits that individuals who feel emotionally secure are more willing to express care for others, thereby promoting prosocial behavior [25].

Although individuals with a lower sense of security may be less inclined to trust others and tend to rely on positive feedback to validate their self-worth [55], a higher level of moral identity can provide essential guidance for their actions. This encourages them to display more prosocial behavior in social interactions to enhance their sense of security and social connection. It is noteworthy that, as shown in Figure 2, although the slope for students with a low sense of security was higher, their level of prosocial behavior consistently remained lower than that of students with a high sense of security. This finding supports the conclusion that sense of security is beneficial for promoting individual mental health [56].

Therefore, enhancing individuals’ sense of security can not only reinforce their moral identity but also effectively promote prosocial behavior, thereby providing strong support for cultivating a more harmonious and supportive social environment. To enhance the sense of security among college students, educators should guide them to develop a correct self-understandings, assist them in comprehending and accepting their own emotions and needs, and help them establish a positive self-concept along with effective emotion-focused coping mechanism. For college students themselves, they can gain emotional support and sense of security by expressing their feelings openly and trying to listen to and empathize with others.

### 5.4. Limitations and Future Directions

Firstly, this study used a cross-sectional design; thus, future research should employ experimental designs or longitudinal studies to more accurately reveal the causal relationships between these variables. Secondly, although we have controlled for additional variables such as age and gender in our analysis, other confounding variables like economic status may still have potential effects on our study. For instance, individual personality traits might influence the relationships between empathy, moral identity, and prosocial behavior. Future research should consider and control for these potential variables more thoroughly to enhance the study’s internal validity. Third, the geographical scope of sample selection was limited to two public teacher training universities in Jiangsu and Heilongjiang provinces. Within the context of Chinese culture, teacher training programs are more popular among female students, resulting in a higher proportion of female students in this study group. Future research should consider collecting samples from a broader geographical area, as well as from various types of institutions and disciplines, to enhance the representativeness and generalizability of the study. Fourth, this study primarily relied on self-reported data, which may introduce bias into the results. To address this limitation, future research could incorporate a variety of data sources, such as behavioral observations and physiological measures, to provide a more comprehensive and objective assessment of these psychological variables.

## 6. Conclusions

In conclusion, this study enriches the theoretical framework of the relationships between empathy, moral identity, sense of security, and prosocial behavior. It also provides valuable insights for educational practice. Educators can effectively encourage prosocial behavior among college students by nurturing their empathy, enhancing their moral identity, and establishing a learning environment rich in a sense of security.

## Figures and Tables

**Table 1:** Descriptive statistics and correlations among variables (N = 840).

Variable	*M*	*SD*	1	2	3	4	5	6
1. Gender	0.83	0.38	1					
2. Age	19.41	0.93	−0.04	1				
3. Empathy	46.72	7.07	0.08 *	0.01	1			
4. Prosocial behavior	20.86	4.76	0.13 **	0.05	0.43 **	1		
5. Moral identity	36.79	6.40	0.17 **	0.10 **	0.49 **	0.65 **	1	
6. Sense of security	49.52	13.01	0.03	0.06	0.09 **	0.35 **	0.24 **	1

Note: Gender: 0 = male, 1 = female; *M* = mean value; *SD* = standard deviation. * *p*  <  0.05, ** *p*  <  0.01.

**Table 2:** Mediation effect test (N = 840).

Dependent Variable	Independent Variable	*R^2^*	*F*	*β*	*SE*	*t*
Moral identity	Gender	0.26	99.66 ***	0.14	0.08	4.64 ***
	Age			0.10	0.03	3.24 **
	Empathy			0.47	0.03	15.95 ***
Prosocial behavior	Gender	0.20	67.59 ***	0.10	0.08	3.26 **
	Age			0.05	0.03	1.44
	Empathy			0.42	0.03	13.50 ***
Prosocial behavior	Gender	0.44	160.83 ***	0.02	0.07	0.86
	Age			−0.01	0.03	−0.40
	Empathy			0.15	0.03	5.01 ***
	Moral identity			0.57	0.03	18.84 ***

Note: ** *p*  <  0.01,*** *p*  <  0.001.

**Table 3:** Moderated mediation model test (N = 840).

Dependent Variable	Independent Variable	*R^2^*	*F*	*β*	*SE*	*t*
Prosocial behavior	Gender	0.50	139.88 ***	0.02	0.03	0.96
	Age			−0.02	0.03	−0.83
	Empathy			0.12	0.03	4.32 ***
	Moral identity			0.51	0.03	17.40 ***
	Sense of security			0.26	0.03	9.77 ***
	Moral identity × Sense of security			−0.14	0.02	−6.49 ***

Note: *** *p * <  0.001.

## Data Availability

The data presented in this study are available on request from the corresponding author (the data are not publicly available due to privacy or ethical restrictions).
